# Case Report and Retrospective Review of *Acanthamoeba* Encephalitis and Chronic Lymphocytic Leukemia, United States

**DOI:** 10.3201/eid3209.260790

**Published:** 2026-09

**Authors:** Michele Persico, Julia C. Haston, Erin Imada, Jennifer Kasten, Cederic Pimentel, Jeffrey M. Collins

**Affiliations:** Emory University, Atlanta, Georgia, USA (M. Persico, C. Pimentel, J.M. Collins); Centers for Disease Control and Prevention, Atlanta (J.C. Haston, E. Imada, J. Kasten)

**Keywords:** Acanthamoeba, meningitis/encephalitis, chronic lymphocytic leukemia, protozoal infections, parasites, waterborne illness, survival, United States

## Abstract

We describe a case of *Acanthamoeba* encephalitis with chronic lymphocytic leukemia (CLL) in the United States and summarize the Free-Living Ameba database to highlight CLL as the most common underlying malignancy for *Acanthamoeba* infections. CLL patients are more likely to have improved survival and nondisseminated cutaneous disease than those with other cancers.

*Acanthamoeba* is a free-living amoeba (FLA) transmitted via inhalation or direct contact with the eyes or skin (*1*) that can cause keratitis and granulomatous amebic encephalitis (GAE) (*2*). GAE occurs most often in immunocompromised patients; risk factors include hematologic malignancy and immunosuppressive drugs (*2*). Chronic lymphocytic leukemia (CLL), the most common leukemia (*3*), can lead to immune dysfunction from immunosuppressive treatments and hypogammaglobulinemia, as well as impairments in complement activity, cell-mediated immunity, and neutrophil function (*4*). The 5-year risk for severe infections in CLL is as high as 57% when IgG levels are low (*5*). Ibrutinib, a Bruton tyrosine-kinase inhibitor (*6*), is an immunomodulatory drug used for CLL treatment that increases the risk for invasive infections up to 11.4% during the first year of treatment through innate immune system impairment (*7*–*9*). We report a case of *Acanthamoeba* GAE in a patient with CLL managed with ibrutinib. Using a retrospective analysis of data from the Centers for Disease Control and Prevention (CDC) Free-Living Ameba database (Appendix), we sought to determine the frequency of *Acanthamoeba* infections among patients with CLL and to compare clinical characteristics with those of patients without CLL.

## The Study

A 71-year-old man with CLL who was receiving ibrutinib sought care for fever, encephalopathy, and focal right-sided weakness. Neuroimaging showed a rapidly enlarging left frontal lesion with surrounding edema; test results for bacterial, viral, and parasitic pathogens were unremarkable (Appendix Table). Brain biopsy demonstrated necrosis, inflammation, and rare Periodic acid-Schiff–positive organisms (Figure). CDC testing confirmed *Acanthamoeba* infection. Despite initiation of azithromycin, sulfadiazine, flucytosine, fluconazole, and miltefosine, the patient died 23 days after symptom onset.

**Figure:**
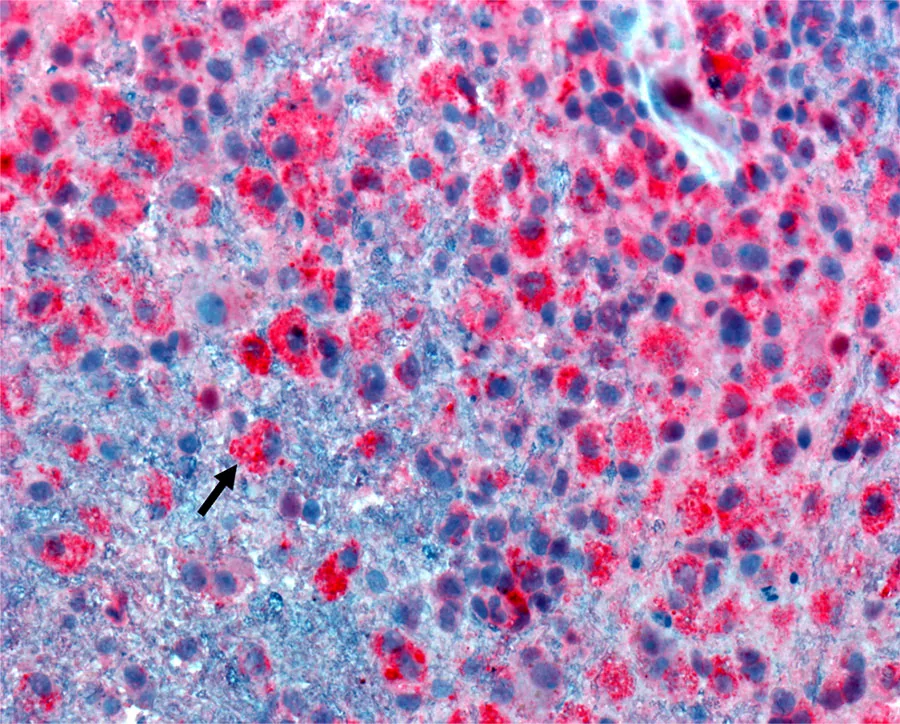
*Acanthamoeba* immunoassay from study of *Acanthamoeba* encephalitis and chronic lymphocytic leukemia, United States. Arrow indicates free-living amebic trophozoites of small to medium size that have rare double-walled cysts and show immunoreactivity to an *Acanthamoeba* spp. immunoassay.

To further investigate the possible role of CLL in this patient’s course, we examined all US cases of *Acanthamoeba* infection reported to the CDC FLA database during 1956–2023 (Appendix). This activity was reviewed by CDC, deemed not research, and was conducted consistent with applicable federal law and CDC policy (see e.g., 45 C.F.R. part 46, 21 C.F.R. part 56; 42 U.S.C. §241(d); 5 U.S.C. §552a; 44 U.S.C. §3501 et seq.). 

The mean age of diagnosis was 48 years for all case-patients with *Acanthamoeba* infection and 68 years in the CLL subgroup (Table). Among the 16 *Acanthamoeba* cases in CLL patients with known sex, 14 occurred in male patients and 2 in female patients, compared with 118 male patients and 173 female patients in cases without CLL. Fifty-six patients had >1 malignancy at the time of *Acanthamoeba* diagnosis: 16 had CLL alone, 1 had CLL and non-Hodgkin lymphoma, and 30 had >1 non-CLL hematologic malignancy (5 with acute lymphoblastic leukemia, 7 with acute myeloid leukemia, 2 with chronic myeloid leukemia, and 13 with lymphoma). Twelve had >1 nonhematologic malignancy.

**Table:** Clinical and demographic characteristics of *Acanthamoeba* cases in the United States during 1956–2023*

Characteristic	Total no. patients	No. (%) patients				
		Any cancer, n = 56	No cancer, n = 137	CLL, n = 17	Other hematologic malignancy, n = 31	Nonhematologic malignancy, n = 12
Age group, y						
0–17	11	2 (3.6)	9 (6.6)	0	2 (6.5)	0
18–34	36	5 (8.9)	31 (22.6)	0	5 (16.1)	1 (8.3)
35–64	105	25 (44.6)	80 (58.4)	6 (35.3)	16 (51.6)	4 (33.3)
>65	36	23 (41.1)	13 (9.5)	11 (64.7)	7 (22.6)	7 (58.3)
Unknown	5	1 (1.8)	4 (2.9)	0	1 (3.2)	0
Sex						
M	132	37 (66.1)	95 (69.3)	14 (82.4)	17 (54.8)	9 (75)
F	57	17 (30.4)	40 (29.2)	2 (11.8)	13 (41.9)	3 (25)
Unknown	4	2 (3.6)	2 (1.5)	1 (5.9)	1 (3.2)	0
Ethnicity/race						
Hispanic	21	5 (8.9)	16 (11.7)	0	4 (12.9)	1 (8.3)
NH White	24	13 (23.2)	11 (8)	9 (53)	3 (9.7)	2 (16.7)
NH Asian	3	0	3 (2.2)	0	0	0
NH Black	6	0	6 (4.4)	0	0	0
NH American Indian or Alaskan Native	1	0	1 (0.7)	0	0	0
Other	51	8 (14.3)	43 (31.4)	2 (11.8)	5 (16.1)	2 (16.7)
Unknown	87	30 (53.6)	57 (41.6)	6 (35.3)	19 (61.3)	7 (58.3)
Survived						
Y	32	11 (19.6)	21 (15.3)	8 (47.1)	2 (6.5)	1 (8.3)
N	133	34 (60.7)	99 (72.3)	3 (17.6)	25 (80.6)	10 (83.3)
Unknown	28	11 (19.6)	17 (12.4)	6 (35.3)	4 (12.9)	1 (8.3)
Disease manifestation						
GAE only	78	20 (35.7)	58 (42.3)	3 (17.6)	12 (38.7)	7 (58.3)
Cutaneous only	19	8 (14.3)	11 (8)	5 (29.4)	3 (9.7)	0
Rhinosinusitis only	8	1 (1.8)	7 (5.1)	1 (5.9)	0	0
Disseminated with GAE	58	19 (33.9)	39 (28.5)	5 (29.4)	12 (38.7)	4 (33.3)
Disseminated without GAE	30	8 (14.3)	22 (16.1)	3 (17.6)	4 (12.9)	1 (8.3)

*The total number of patients with cancer was 56. The sum of patients represented in the CLL, other hematologic malignancy, and nonhematologic malignancy categories is 60; several patients had >1 malignancy and are represented in multiple categories. CLL, chronic lymphocytic leukemia; GAE, granulomatous amebic encephalitis; NH, non-Hispanic.

Of 17 patients with CLL, 8 had central nervous system (CNS) involvement, including 3 with GAE alone and 5 with GAE and disseminated disease. In comparison, CNS involvement was seen among 24 of 31 with non-CLL hematologic malignancies and 11 out of 12 of those with non-hematologic malignancies. Nine of 17 CLL patients had non-CNS *Acanthamoeba* infections: 5 with cutaneous disease, 1 with rhinosinusitis, and 3 with non-CNS disseminated disease.

Of the 17 CLL cases, 8 survived, 3 died, and 6 had an unknown survival status. The 3 patients with CLL who died all had GAE, whereas 1 of 8 survivors had GAE. Among patients with a non-CLL malignancy and known survival outcome (n = 33), 2 patients survived and 31 died. Neither of the 2 non-CLL survivors had GAE; GAE was present in 28 of 31 non-CLL patients who died. *Acanthamoeba* infection in patients with CLL was associated with lower odds for death than non-CLL malignancies (odds ratio [OR] 0.03 [95% CI 0.003–0.18]; p<0.0001); that OR was likely a result of the lower frequency of GAE among CLL patients. GAE was associated with markedly greater odds of death in patients with any type of cancer (OR 68.6 [95% CI 9.0–2,075.9]; p<0.0001).

## Conclusions

We investigated the frequency of CLL among patients with *Acanthamoeba* infection and its effect on survival. CLL was the most prevalent malignancy among patients with *Acanthamoeba* infection. Of note, a lower number of *Acanthamoeba* cases occurred in persons with hematologic malignancies that are generally associated with a higher risk for opportunistic infections, including acute myeloid leukemia and acute lymphoblastic leukemia. Those findings suggest that unique immune impairments in CLL might predispose to disease from *Acanthamoeba*. Potential mechanisms include hypogammaglobulinemia and neutropenia (*10*), which could potentially be mitigated by immunoglobulin replacement. Our case study demonstrated that, although GAE most often has a subacute clinical course, it can have a rapid onset and progression. In addition, we showed that CLL and ibrutinib exposure could be important risk factors for disease caused by *Acanthamoeba.*

The clinical course and mortality rate of nonkeratitis *Acanthamoeba* infections depend on the organ system involved. GAE has the highest mortality rate, exceeding 90%, whereas death from cutaneous *Acanthamoeba* infection or rhinosinusitis is less common (*11*). We found that patients with CLL had significantly higher odds of survival compared with patients who had other hematologic malignancies or solid tumors; that result was partially explained by the relatively high proportion of *Acanthamoeba* infections in CLL patients without CNS involvement. In our study, 53% of patients with CLL had non-CNS *Acanthamoeba* infections, whereas 30% of the overall cohort of patients had non-CNS *Acanthamoeba* infections (Table). Previous studies have reported lower mortality rates among those who did not have CNS involvement (*2*). The older age in the CLL group likely reflected the higher median age, 64 years, at CLL diagnosis (*12*). Patients with CLL are predominantly male (66%–69%) (*12*), which is similar to the overall sex distribution among *Acanthamoeba* cases in the FLA database.

Including the patient in our report, of the 18 reported patients with CLL, >4 patients have been treated with ibrutinib since it was introduced to the US market in 2012. Similar to our case, 2 other cases describe a more acute course, in which death occurred 4–14 days after the patients initially sought care at the hospital (*13*,*14*). That finding was unusual because GAE is typically considered a subacute to chronic form of encephalitis (*2*). Given the small number of cases reported, it is unclear whether the more acute clinical course can be attributed to CLL or immunosuppressive treatment. Further monitoring could determine whether ibrutinib may increase the risk for *Acanthamoeba* infection or death.

Our cross-sectional analysis of US disease surveillance data had limitations. For patients with a cancer diagnosis, detailed clinical and laboratory data, as well as treatment history and survival time, were not routinely collected in the CDC FLA database. In addition, information on the method of *Acanthamoeba* diagnosis, treatment, and time to death after diagnosis was not generally available. Last, the CDC FLA database includes *Acanthamoeba* cases from the United States only, which limited generalizability of our results.

Since 1956, a total of 18 *Acanthamoeba* infections have been identified in patients with CLL, including the case we reported here. CLL is the most common malignancy among patients with invasive *Acanthamoeba* infections. CLL patients with *Acanthamoeba* infections had lower rates than patients with other malignancies, which could be attributed to a greater likelihood of cutaneous disease and lower rates of CNS involvement. Further monitoring can elucidate the clinical factors and treatments mediating the association between CLL and *Acanthamoeba* infection.

Appendix Additional information from case report and retrospective review of *Acanthamoeba* encephalitis and chronic lymphocytic leukemia, United States.

## References

[1] Visvesvara GS, Moura H, Schuster FL. Pathogenic and opportunistic free-living amoebae: *Acanthamoeba* spp., *Balamuthia mandrillaris, Naegleria fowleri*, and *Sappinia diploidea.* FEMS Immunol Med Microbiol. 2007;50:1–26. 10.1111/j.1574-695X.2007.00232.x 17428307

[2] Haston JC, O’Laughlin K, Matteson K, Roy S, Qvarnstrom Y, Ali IKM, et al. The epidemiology and clinical features of non-keratitis *Acanthamoeba* infections in the United States, 1956–2020. Open Forum Infect Dis. 2023;10:ofac682. 10.1093/ofid/ofac682 36655187 PMC9835757

[3] Fedele PL, Opat S. Chronic lymphocytic leukemia: time to care for the survivors. J Clin Oncol. 2024;42:2005–11. 10.1200/JCO.23.02738 38489567

[4] Morrison VA. Management of infectious complications in patients with chronic lymphocytic leukemia. Hematology (Am Soc Hematol Educ Program). 2007;2007:332–8. 10.1182/asheducation-2007.1.332 18024648

[5] Molica S, Levato D, Levato L. Infections in chronic lymphocytic leukemia. Analysis of incidence as a function of length of follow-up. Haematologica. 1993;78:374–7. 8175032

[6] Cameron F, Sanford M. Ibrutinib: first global approval. Drugs. 2014;74:263–71. 10.1007/s40265-014-0178-8 24464309

[7] Fiorcari S, Maffei R, Vallerini D, Scarfò L, Barozzi P, Maccaferri M, et al. BTK inhibition impairs the innate response against fungal infection in patients with chronic lymphocytic leukemia. Front Immunol. 2020;11:2158. 10.3389/fimmu.2020.02158 32983178 PMC7485008

[8] Stephens DM, Byrd JC. How I manage ibrutinib intolerance and complications in patients with chronic lymphocytic leukemia. Blood. 2019;133:1298–307. 10.1182/blood-2018-11-846808 30642919 PMC6428663

[9] Varughese T, Taur Y, Cohen N, Palomba ML, Seo SK, Hohl TM, et al. Serious infections in patients receiving ibrutinib for treatment of lymphoid cancer. Clin Infect Dis. 2018;67:687–92. 10.1093/cid/ciy175 29509845 PMC6093991

[10] Bloch KC, Schuster FL. Inability to make a premortem diagnosis of *Acanthamoeba* species infection in a patient with fatal granulomatous amebic encephalitis. J Clin Microbiol. 2005;43:3003–6. 10.1128/JCM.43.6.3003-3006.2005 15956445 PMC1151932

[11] Siddiqui R, Khan NA. Biology and pathogenesis of *Acanthamoeba.* Parasit Vectors. 2012;5:6. 10.1186/1756-3305-5-6 22229971 PMC3284432

[12] Shanafelt TD, Rabe KG, Kay NE, Zent CS, Jelinek DF, Reinalda MS, et al. Age at diagnosis and the utility of prognostic testing in patients with chronic lymphocytic leukemia. Cancer. 2010;116:4777–87. 10.1002/cncr.25292 20578179 PMC3902481

[13] Crothers JW, Hsu L, Marty FM. Fulminant *Acanthamoeba castellanii* encephalitis in an ibrutinib-treated patient. Open Forum Infect Dis. 2020;7:ofaa025. 10.1093/ofid/ofaa025 32055639 PMC7008473

[14] Voshtina E, Huang H, Raj R, Atallah E. Amebic encephalitis in a patient with chronic lymphocytic leukemia on ibrutinib therapy. Case Rep Hematol. 2018;2018:6514604. 10.1155/2018/6514604 30155323 PMC6092972

