# Supplementary material for: Case Report and Retrospective Review of Acanthamoeba Encephalitis and Chronic Lymphocytic Leukemia, United States
Source: Emerg Infect Dis. 2026 Sep;32(9):1483–6. doi: 10.3201/eid3209.260790 (PMC13555711; doi:10.3201/eid3209.260790)
Supplement: Supplementary file 1 [file 26-0790-Techapp-s1.pdf]

Article DOI: https://doi.org/10.3201/eid3209.260790

EID cannot ensure accessibility for supplementary materials supplied by authors. Readers who have difficulty accessing supplementary content should contact the authors for assistance.

Case Report and Retrospective Review of *Acanthamoeba* Encephalitis and Chronic Lymphocytic Leukemia, United States

Appendix

Methods

We examined data describing all US cases of *Acanthamoeba* infection reported to the Centers for Disease Control and Prevention (CDC) free-living amoeba (FLA) database between 1956 and 2023 (Table 1). The CDC FLA database is a national case surveillance system to collect information about confirmed FLA infections in the United States, including those caused by *Acanthamoeba* species (non-keratitis infections), *Balamuthia mandrillaris*, and *Naegleria fowleri*. Cases in the system are reported by clinicians, state and local health departments, or found through published case reports. The reporting of FLA infections is not mandatory. In 2011, the Council for State and Territorial Epidemiologists (CSTE) established a position statement that outlined case definition criteria, which were most recently revised in 2017. The CDC FLA database collects data on demographic, clinical features, medical history, exposures, treatment, diagnosis, and outcomes. These data are reported voluntarily by state health departments through the System for Enteric Disease Response, Investigation, and Coordination (SEDRIC) platform. For this analysis, we used demographic, medical history, clinical features, diagnosis, and outcome variables of interest.

We extracted all 193 *Acanthamoeba* cases from the CDC's FLA database from 1956 to 2023. We excluded the case described in the case report from the analysis as it occurred outside the included study years. We categorized all cases into age groups, sex, ethnicity, race, and disease presentation. Disease presentation categories included granulomatous encephalitis (GAE) only, cutaneous only, rhinosinusitis only, dissemination with GAE, and dissemination without

GAE. Cases were categorized as GAE only when there was evidence of brain infection and no evidence of cutaneous, rhinosinusitis, or disseminated involvement. Cases were categorized as cutaneous only when there was evidence of cutaneous infection and no evidence of brain, rhinosinusitis, or disseminated involvement. Cases were categorized as rhinosinusitis only when there was evidence of rhinosinusitis infection and no evidence of brain, cutaneous, or disseminated involvement. Cases were categorized as disseminated without GAE when there was evidence of disseminated infection in multiple sites and no evidence of brain involvement. Cases were categorized as disseminated with GAE when there was evidence of brain infection plus one or more additional sites of involvement. Next, cases were categorized into clinical characteristics of history of cancer, chronic lymphocytic leukemia (CLL), other hematologic malignancy, and non-hematologic malignancy. Missing or unknown responses for CLL and hematologic malignancy were classified as 'No'.

Statistical analyses were performed in R version 4.4.1. All statistical analyses were replicated by two authors to ensure accuracy. Odds ratios were calculated using the epitools package, with p-values calculated using a Fisher Exact Test.

Appendix Table. Testing for infectious (bacterial, viral, and parasitic pathogens) and non-infectious (autoimmune, neoplastic) etiologies in the case presented in this study

Serum test	Result	Cerebrospinal fluid test	Result
Cryptococcal antigen	Negative	Cryptococcal antigen	Negative
(1,3)-Beta-D-Glucan (Fungitell)	Negative	Venereal Disease Research Laboratories	Non-reactive
Schistosoma IgG	Negative	Herpes simplex virus PCR	Negative
Bartonella Henselae IgG and IgM	Negative	Varicella zoster virus PCR	Negative
Coxiella Burnetii IgG Phase I and II,	Negative	*Toxoplasma gondii* PCR	Not detected
Borrelia burgdorferi IgG and IgM	Negative	Meningitis/encephalopathy PCR panel (include Epstein-Barr virus, cytomegalovirus, E. Coli K1, Haemophilus influenzae, Listeria monocytogenes, Neisseria meningitidis, Streptococcus agalactiae, Streptococcus pneumoniae, Enterovirus, human herpesvirus 6, and Human Parechovirus)	Not detected
Human immunodeficiency virus antigen and antibodies	Negative	CSF cytology	Negative for malignant cells
West-Nile polymerase chain reaction (PCR)	Not detected	Leukemia/Lymphoma profile	Absent abnormal hematolymphoid cell populations.
Bacterial culture	No growth	Autoimmune encephalopathy panel (include NMDA receptor antibody, LGI-1IgG Antibody CBA, CASPR2-IgG Antibody CBA, Anti-GAD65, GABA-B-Receptor Antibody CBA, AMPA-R antibody CBA, ANNA-1, ANNA-2, ANNA-3, Anti-Glial Nuclear, Anti-Purkinje Cell Cytoplasm 1, Anti-Purkinje Cell Cytoplasm 2, Anti-Purkinje Cell Cytoplasm TR, Anti-Amphiphysin, Anti-Collapsin Resp Med Protein 5, IgLON5 IFA, DDPX Antibody IFA, GFAP IFA, mGLUR1 Antibody IFA, Neurochondrin IFA)	Negative
Fungal culture	No growth		
Acid-fast bacilli culture	No growth		
